# Depression and anxiety in adolescents with cystic fibrosis in Brazil: prevalence, stability over time, and relationship with treatment adherence

**DOI:** 10.36416/1806-3756/e20240416

**Published:** 2025-05-21

**Authors:** Tatiana Rozov, Marcos Tadeu Nolasco da Silva, Maria Ângela Gonçalves Oliveira Ribeiro, Neiva Damaceno, Maria Angelica Pinheiro Santos Santana, Paulo Jose Cauduro Marostica, Roberta de Cássia Nunes Cruz Melotti, Valeria de Carvalho Martins, Carlos Antônio Riedi, Edna Lucia Santos de Souza, Norberto Ludwig, Rodrigo Abensur Athanazio, Francyelly Wisnievski Yamamoto, Lusmaia Damaceno Camargo Costa, Sonia Mayumi Chiba, Izabela Sad, José Dirceu Ribeiro, Alexandra L. Quittner, Brazilian Anxiety, Depression and Adherence Study Group (BADAS)1

**Affiliations:** 1. Universidade de São Paulo, São Paulo (SP) Brasil.; 2. Universidade Estadual de Campinas, Campinas (SP) Brasil.; 3. Irmandade da Santa Casa de Misericórdia de São Paulo, São Paulo (SP) Brasil.; 4. Hospital Otavio Mangabeira, Salvador (BA) Brasil.; 5. Universidade Federal do Rio Grande do Sul, Porto Alegre (RS) Brasil.; 6. Centro de Referência em Fibrose Cística do Espírito Santo, Vitória (ES) Brasil.; 7. Universidade Federal do Pará, Belém (PA) Brasil.; 8. Universidade Federal do Paraná, Curitiba (PR) Brasil.; 9. Universidade Federal da Bahia, Salvador (BA) Brasil.; 10. Hospital Infantil Joana de Gusmão, Florianópolis (SC) Brasil.; 11. Universidade Estadual Paulista, Botucatu (SP) Brasil.; 12. Universidade Federal de Goiás, Goiânia (GO) Brasil.; 13. Universidade Federal de São Paulo, São Paulo (SP) Brasil.; 14. Instituto Fernandes Figueira, Rio de Janeiro (RJ) Brasil.; 15. Joe DiMaggio Cystic Fibrosis, Pulmonary & Sleep Center, Holywood (FL) USA.

**Keywords:** Cystic fibrosis, Anxiety, Depression, Treatment adherence and compliance, Adolescent

## Abstract

**Objective:**

Depression and anxiety have been documented in people with cystic fibrosis (CF), jeopardizing treatment adherence. To date, no studies have assessed the prevalence of psychosocial issues in adolescents with CF in Brazil. We sought to assess the prevalence of depression and anxiety in adolescents with CF in Brazil, as well as the impact of depression and anxiety on treatment adherence.

**Methods:**

This was a multicenter, prospective, observational, longitudinal study conducted between 2017 and 2019 at 14 CF referral centers in Brazil. We used standardized tools such as the nine-item Patient Health Questionnaire (for depression), the seven-item Generalized Anxiety Disorder scale (for anxiety), and the eight-item Morisky Medication Adherence Scale (for treatment adherence) in order to collect data on 218 CF patients at two different time points.

**Results:**

The prevalence of depression was 19.1% at time point 1 and 15.4% at time point 2. The prevalence of anxiety was 19.1% at time point 1 and 18.0% at time point 2. Depression and anxiety were significantly higher in female patients and lower in those who underwent home physiotherapy or had psychological support. Significant correlations were found between depression and anxiety at both time points, the associations being strongest at time point 1 (r = 0.68; p < 0.001). Most (66.7%) of the study participants reported low adherence to treatment, and the remainder reported either average adherence (in 28%) or high adherence (in 5.3%). Depression and anxiety showed inverse correlations with treatment adherence.

**Conclusions:**

The prevalence of depression and anxiety in adolescents with CF in Brazil appears to be similar to that reported in other countries, being higher in females and lower in those undergoing home physiotherapy or receiving psychological care. Depression and anxiety appear to correlate with lower treatment adherence. Treating psychosocial issues may effectively improve rates of treatment adherence in adolescents with CF.

## INTRODUCTION

In a recent systematic review and meta-analysis including 26 studies conducted across European and North American countries, the mean prevalence of depression in people with cystic fibrosis (CF) was found to be 18.7% in North America and 13.3% in Europe, and the mean prevalence of anxiety was found to be 23.6% in North America and 26.8% in Europe.^1^ Additionally, studies have shown that depression and anxiety are associated with a higher number of pulmonary exacerbations, worse adherence to prescribed treatments, missed clinic visits, and worse health-related quality of life (HRQoL).^2^ The objective of the aforementioned systematic review and meta-analysis was to determine the level of anxiety and depression among people with CF worldwide.^1^ However, data from Latin American countries such as Brazil were not included in that study.^3^ ^-^ ^5^ There are approximately 5,128 CF patients registered with the *Grupo Brasileiro de Estudos de Fibrose Cística* (Brazilian Cystic Fibrosis Study Group); of those, 1,590 (31%) are adolescents.^6^

CF is a progressive lung disease that affects multiple organ systems and leads to frequent pulmonary exacerbations; worse energy and physical functioning; difficulty gaining and maintaining weight; and an increased number of days in hospital. Disease progression increases limitations in daily activities, treatment burden, and uncertainty about the future. These stressors are associated with increased symptoms of depression and anxiety, as well as with medical traumatic stress and worsening HRQoL.^3^ ^,^ ^7^ ^-^ ^11^ There are currently no data on the prevalence of depression and anxiety in CF patients or the impact of depression and anxiety on CF management in Brazil or other Latin American countries. 

Most of the CF patients followed at referral centers in Brazil have class I or II mutations in the cystic fibrosis transmembrane conductance regulator (*CFTR*) gene, all of which are associated with severe forms of CF that lead to multisystem involvement.^6^ Disease severity has been correlated with an increased prevalence of depression and anxiety in some studies. Graziano et al.^5^ found a high prevalence of depression as reported by patients with CF and their parents (adolescents, 37%; adults, 45%; and parents, 49%). Symptoms of anxiety were also highly prevalent (adolescents, 48%; adults, 46%; and parents, 66%). In a landmark study including more than 6,000 adolescents and adults with CF and over 4,200 caregivers across nine countries, symptoms of depression and anxiety were found to be 2-3 times more common in CF patients than in community samples.^3^

Quittner et al.^11^ found that 19% of adolescents with CF, 29% of adults with CF, 34% of mothers of patients with CF, and 25% of fathers of patients with CF were above the clinical cutoff for the Center for Epidemiological Studies Scale for Depression, and 22% of adolescents with CF, 32% of adults with CF, 48% of mothers of patients with CF, and 36% of fathers of patients with CF were above the clinical cutoff for the Hospital Anxiety and Depression Scale. Although a number of studies have been published on rates of depression and anxiety in Europe and North America, studies of mental health in countries with higher rates of economic and cultural inequality*,* such as Brazil, are scarce, the exception being a study performed in Turkey and reporting higher prevalences of depression and anxiety in children and adolescents with CF in comparison with a control group.^12^

Following the publication of the aforementioned landmark study,^3^ the European Cystic Fibrosis Society and the Cystic Fibrosis Foundation developed international guidelines on mental health screening and treatment, recommending close patient monitoring; annual screening of depression and anxiety; and adoption of appropriate prevention and intervention strategies.^3^ ^,^ ^11^ ^,^ ^13^ New CF-specific cognitive behavioral therapy interventions have been developed in the USA and are being disseminated in Europe and Australia.^14^ ^-^ ^17^

The objectives of the present epidemiological study were as follows: to assess the prevalence of depression and anxiety in adolescents with CF followed at referral centers in Brazil; to examine the stability of depression and anxiety scores over time; to assess self-reported adherence to CF treatments; and to evaluate the relationship of depression and anxiety with sociodemographic variables and rates of treatment adherence. 

## METHODS

### Study design and setting

This was a multicenter, prospective, observational, longitudinal study conducted at 14 CF referral centers in Brazil, with data collection occurring between 2017 and 2019. All of the patients followed at the 14 participating centers were invited to participate in the study, as were their caregivers. The inclusion criteria were being in the 10- to 19-year age bracket and having a diagnosis of CF confirmed by two or more sweat chloride measurements ≥ 60 mEq/L and/or two disease-causing mutations in the *CFTR* gene. A total of 218 patients were enrolled in the study, corresponding to 13.7% of 1,590 adolescents included in the Brazilian Cystic Fibrosis Registry. All participating patients were asked if they were undergoing psychological/psychiatric treatment and if they were taking any psychoactive drugs. 

### Procedures

During a routine outpatient visit, the study participants completed questionnaires assessing depression, anxiety, and treatment adherence, as well as completing a sociodemographic form. The stability of scores over time was assessed at two different time points, at least three months apart. 

The study was approved by the institutional review boards of all participating centers including the coordinating center (Protocol no. 1.851.482). The study was conducted in accordance with the ethical standards established in Brazilian National Health Council Resolution no. 466/2012. 

Written informed consent was obtained from all the participants. In the case of participants < 18 years of age, consent was obtained from their caregivers. 

### Measures

#### Demographic and medical variables

We collected data on the following demographic variables: sex; age; age at diagnosis of CF; and level of education. In addition, we collected data on the following anthropometric and medical variables: height for age; BMI; Shwachman-Kulczycki scores; FEV_1_; FVC; FEV_1_/FVC; FEF_25-75%_; use of oxygen therapy; home physiotherapy; bacterial colonization of the airways; chronic use of azithromycin; pancreatic insufficiency; liver disease; CF-related diabetes; and newborn screening for CF. Furthermore, we collected data on the following psychosocial variables: a diagnosis of anxiety, depression, or both; prior referral for psychological or psychiatric treatment; and use of psychoactive drugs. 

The following patient-reported outcome measures were administered: the nine-item Patient Health Questionnaire (PHQ-9),^18^ ^,^ ^19^ the seven-item Generalized Anxiety Disorder (GAD-7) scale,^19^ and the eight-item Morisky Medication Adherence Scale (MMAS-8),^20^ all of which were used after permission was granted from the original authors. 

#### The PHQ-9

The PHQ-9^18^ ^,^ ^19^ is a self-report measure of depressive symptoms mapping on to the diagnostic criteria for depression. The PHQ-9 was developed by Spitzer et al.^18^ and validated for use by Kroenke et al.^18^ It has extensive evidence of reliability (Cronbach’s alpha = 0.86-0.89), validity, sensitivity, and specificity. Each symptom is rated on a four-point scale ranging from 0 (not at all) to 3 (nearly every day) over the past two weeks; one question asks about suicidal ideation. Depression is categorized as no symptoms (0-4), mild (5-9), moderate (10-14), or severe (> 15). We used a Brazilian Portuguese version of the PHQ-9, validated for use in Brazil by Osório et al.^21^

#### The GAD-7 scale

The GAD-7 scale^19^ is a self-report measure assessing generalized anxiety, with extensive evidence of reliability (Cronbach’s alpha = 0.92) and validity. Each symptom is rated on a four-point scale from 0 (not at all) to 3 (nearly every day) over the past two weeks. Anxiety is categorized as no symptoms (0-4), mild (5-9), moderate (10-14), or severe (> 15). The GAD-7 scale was developed by Spitzer et al.^19^ and validated for use by Kroenke et al.^18^ on the basis of Diagnostic and Statistical Manual of Mental Disorders, fourth edition criteria. It was translated into Portuguese and validated for use in Brazil by the Mapi Research Trust in 2006. 

#### The MMAS-8

The MMAS-8 was administered to assess adherence to prescribed medications and treatments. It is a brief, self-report scale developed by Morisky et al.^21^ and validated for use in Brazil by Oliveira-Filho et al.^22^ Scores range from 0-8, as follows: 8, high adherence; 6-7, average adherence; and < 6, low adherence. MMAS-8 scores were collapsed into two categories: low adherence and average/high adherence. 

### Statistical analysis

Data were collected and entered into a database created with the IBM SPSS Statistics software package, version 26.0 for Windows (IBM Corporation, Armonk, NY, USA). Normality was assessed by the Kolmogorov-Smirnov test. For analysis of associations between continuous and categorical variables, the Mann-Whitney test was used. Associations between categorical variables were assessed by the chi-square test. 

Associations between two continuous variables were evaluated by Pearson’s correlation coefficients. For assessment of agreement between depression and anxiety at two different time points, intraclass correlation coefficients were computed. The level of significance was set at p ≤ 0.05. 

## RESULTS

**Figure 1:**
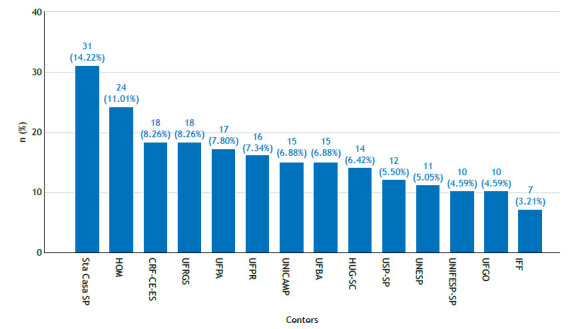
Numbers and proportions of patients from the 14 cystic fibrosis referral centers participating in the present study. Sta Casa SP: *Irmandade da Santa Casa de Misericórdia de São Paulo* (located in the city of São Paulo, Brazil); HOM: *Hospital Otavio Mangabeira* (located in the city of Salvador, Brazil); UFRGS: *Universidade Federal do Rio Grande do Sul* (located in the city of Porto Alegre, Brazil); CRFC-ES: *Centro de Referência em Fibrose Cística do Espírito Santo* (located in the city of Vitória, Brazil); UFPA: *Universidade Federal do Pará* (located in the city of Belém, Brazil); UFPR: *Universidade Federal do Paraná* (located in the city of Curitiba, Brazil); UFBA: *Universidade Federal da Bahia* (located in the city of Salvador, Brazil); UNICAMP: *Universidade Estadual de Campinas* (located in the city of Campinas, Brazil); HJG-SC: *Hospital Joana de Gusmão* - *Santa Catarina* (located in the city of Florianópolis, Brazil); USP: *Universidade de São Paulo* (located in the city of São Paulo, Brazil); UNESP: *Universidade Estadual Paulista* (located in the city of Botucatu, Brazil); UFGO: *Universidade Federal de Goiás* (located in the city of Goiânia, Brazil); UNIFESP: *Universidade Federal de São Paulo* (located in the city of São Paulo, Brazil); and IFF: *Instituto Fernandes Figueira* (located in the city of Rio de Janeiro, Brazil).

A total of 218 adolescents with CF followed at any of 14 CF referral centers in Brazil participated in the study. Figure 1 shows the numbers and proportions of patients from each center. 

### Characteristics of the study population

A nationally representative sample was recruited for the present epidemiological study. Overall, girls were significantly older; had significantly lower FEV_1_, FEV_1_/FVC, and FEF_25-75%_; and had been using azithromycin for longer. No significant differences between boys and girls were found for the remaining variables (Table 1). 

**Table 1:** General characteristics of the study population, by sex.^a^

	Total sample (N = 218)	Males (n = 101)	Females (n = 117)	p
Age, years	14.86 [9.90-20.83]	14.16 [10.15-20.77]	15.52 [9.90-20.83]	0.03*
Age at diagnosis of CF, years	1.67 [0.02-19.36]	1.09 [0.04-15.86]	2.70 [0.02 - 19.36]	0.14*
Completed high school	40 (18.9%); 95% CI, 14.2-24.7	18 (17.8%); 95% CI, 11.6-26.4	22 (19.6%); 95% CI, 13.3-27.9	0.71**
Short stature	28 (13.3%); 95% CI, 9.3-18.5	13 (13.3%); 95% CI, 7.9-21.4	15 (13.3%); 95% CI, 8.2-20.7	0.99**
Malnutrition	40 (19.0%); 95% CI, 14.2-24.8	20 (20.4%); 95% CI, 13.6-29.4	20 (17.7%); 95% CI, 11.8-25.8	0.62**
Shwachman-Kulczycki score	80 [40-100]	80 [45-100]	75 [40-100]	0.17*
FEV_1_, % predicted	78 [17-143]	84 [17-143]	66.75 [17-121]	0.02*
FVC, % predicted	87 [26.80-141.00]	91 [32-147]	85 [26.8-129]	0.07*
FEV_1_/FVC	84.77 [13.82-114.76]	87.88 [43.82-114.76]	83.11 [54.72-111.96]	0.01*
FEF_25-75%_	56 [6-210]	66 [6-210]	42.5 [6-135]	0.01*
Pancreatic insufficiency	174 (80.9%); 95% CI, 77.1-85.6	80 (79.2%); 95% CI, 70.3-86.0	94 (82.5%); 95% CI, 74.4-88.3	0.54**
Liver disease	44 (21.9%); 95% CI, 16.7-28.1	21 (21.9%); 95% CI, 14.8-31.1	23 (21.9%); 95% CI, 15.1-30.7	0.99**
Diabetes	22 (10.7%); 95% CI, 7.2-15.7	8 (8.2%); 95% CI, 4.2-15.4	14 (12.9%); 95% CI, 7.9-20.6	0.28**
Chronic airway colonization	142 (71.4%); 95% CI, 64.7-77.2	64 (67.4%); 95% CI, 57.4-76.0	78 (75.0%); 95% CI, 65.9-82.3	0.23**
Chronic airway colonization with *Pseudomonas aeruginosa*	101 (53.2%); 95% CI, 46.1-60.1	49 (59.8%); 95% CI, 48.9-69.7	52 (48.1%); 95% CI, 38.9-57.0	0.15**
Home physiotherapy	158 (75.6%); 95% CI, 69.3-80.9	71 (74.0%); 95% CI, 64.4-81.7	87 (77.0%); 95% CI, 68.4-83.8	0.61**
Home oxygen therapy	12 (5.7%); 95% CI, 3.3-9.7	4 (4.0%); 95% CI, 1.6-9.9	8 (7.2%); 95% CI, 3.7-13.6	0.32**
Azithromycin use	109 (55.9%); 95% CI, 48.9-62.7	41 (46.1%); 95% CI, 36.1-56.4	68 (64.1%); 95% CI, 54.7-72.6	0.01**
Previous psychological evaluation	40 (47.6%); 95% CI, 37.3-58.2	18 (46.1%); 95% CI, 31.7-61.4	22 (48.9%); 95% CI, 35.0-63.0	0.23**
Psychotropic drug use	11 (11.3%); 95% CI, 6.4-19.2	3 (6.8%); 95% CI, 2.3-18.2	8 (1.5%); 95% CI, 7.8-27.0	0.20**

a Data expressed as n (%) or median [IQR]. *Mann-Whitney test (p < 0.05). **Chi-square test (p < 0.05).

### Prevalence of depression and anxiety; stability of anxiety and depression scores over time; and adherence to treatment

PHQ-9 scores showed that 19.1% of the study participants were depressed at time point 1. The prevalence of depression decreased slightly at time point 2, to 15.4%. PHQ-9 scores ranged from 0 to 23 at time point 1 (with a median score of 4) and from 0 to 24 at time point 2 (with a median score of 3). GAD-7 scale scores showed that 19.1% of the study participants suffered from anxiety at time point 1, with a similar proportion (18%) at time point 2. GAD-7 scale scores ranged from 0 to 20 at both time points, with a median score of 4. Girls reported a higher prevalence of depression and anxiety at both time points than did boys. 

With regard to the stability of anxiety and depression scores over time, intraclass correlation coefficients showed a high degree of stability. The level of agreement between depression scores at the two time points was high (intraclass correlation coefficient = 0.81; p < 0.001), with similarly high rates of stability for anxiety (intraclass correlation coefficient = 0.78; p < 0.001).

With regard to the rates of treatment adherence, most (66.7%) of the study participants had low adherence as assessed by the MMAS-8, with 28% reporting moderate adherence and only 5.3% reporting high adherence (Table 2). Treatments were prescribed in accordance with Brazilian guidelines for the diagnosis and treatment of CF.^23^ Primary treatments were provided by the Brazilian Unified Health Care System and included pancreatic enzyme replacement therapy for pancreatic insufficiency; dornase alfa for recurrent pulmonary symptoms; and inhaled tobramycin or colistin for gram-negative bacteria in sputum culture. The triple combination of elexacaftor, tezacaftor, and ivacaftor has yet to be approved for use in Brazil. Scores for depression, anxiety, and treatment adherence are summarized in Table 2. 

**Table 2:** Psychosocial outcomes in the study population, by sex.^a^

	Total sample (N = 218)	Males (n = 101)	Females (n = 117)	p
Depression at T1, PHQ-9	19.1%; 95% CI, 14.4-24.8	11.0%; 95% CI, 6.2-18.6	26.1%; 95% CI, 18.9-34.8	0.005*
Depression at T1, PHQ-9	4 [0-23]	3 [0-22]	5 [0-21]	0.03**
Depression at T2, PHQ-9	15.4%; 95% CI, 11.1-20.9	9.3%; 95% CI, 5.0-16.7	20.7%; 95% CI, 14.2-29.2	0.02*
Depression at T2, PHQ-9	3 [0-24]	3 [0-24]	3 [0-24]	0.46**
Anxiety at T1, GAD-7 scale	19.1%; 95% CI, 14.4-24.8	12.0%; 95% CI, 7.0-19.8	25.2%; 95% CI, 18.2-33.9	0.014*
Anxiety at T1, GAD-7 scale	4 [0-20]	3 [0-20]	6 [0-20]	0.005**
Anxiety at T2, GAD-7 scale	18.0%; 95% CI, 13.4-23.7	10.2%; 95% CI, 5.6-17.8	24.8%; 95% CI, 17.7-33.5	0.006*
Anxiety at T2, GAD-7 scale	4 [0-20]	3 [0-19]	5 [0-20]	0.06**
Treatment adherence, MMAS-8 High Average Low	5.3%; 95% CI, 2.6-10.5 28.0%; 95% CI, 21.2-36.2 66.7%; 95% CI, 58.2-74.1	6.8%; 95% CI, 2.7-16.2 28.8%; 95% CI, 18.6-41.4 64.4%; 95% CI, 51.7-75.4	4.1%; 95% CI, 1.4-11.4 27.4%; 95% CI, 1.5-38.6 68.5; 95% CI, 57.1-78	0.54*
Treatment adherence, MMAS-8	5 [0-8]	5 [1-8]	5 [0-8]	0.93**

T1: time point 1; T2: time point 2; PHQ-9: nine-item Patient Health Questionnaire; GAD-7: seven-item Generalized Anxiety Disorder; and MMAS-8: eight-item Morisky Medication Adherence Scale. ^a^Data expressed as % or median [IQR]. *Chi-square test (p < 0.05). **Mann-Whitney test (p < 0.05).

Some of the independent variables showed an association with psychosocial outcomes. Patients with depression were significantly older at both time points. The age at diagnosis of CF was significantly higher in the patients with anxiety and depression at both time points. Patients undergoing home physiotherapy had lower prevalences of depression and anxiety at both time points, as well as higher treatment adherence rates. Patients without depression (at both time points) or anxiety (at time point 2) had significantly higher Shwachman-Kulczycki general activity domain scores. Patients with high or medium treatment adherence rates had significantly higher Shwachman-Kulczycki radiological findings domain scores (Table 3). 

**Table 3:** Significant associations of depression, anxiety, and treatment adherence with independent variables.^a^

	Depression at T1 (prevalence - %)	Depression at T2 (prevalence - %)	Anxiety at T1 (prevalence - %)	Anxiety at T2 (prevalence - %)	Treatment adherence (prevalence - %) (High/Average vs. Low)
Age, years (median)	15.95 vs. 14.52 p = 0.04**	16.09 vs. 14.53 p = 0.02**	15.87 vs. 14.52 p = 0.10**	15.75 vs. 14.64 p = 0.11**	15.12 vs. 13.85 p = 0.08**
Age at CF diagnosis, years (median)	5.10 vs. 1.24 p = 0.003**	5.10 vs. 1.37 p = 0.02**	4.07 vs. 1.24 p = 0.007**	4.86 vs. 1.24 p = 0.004**	2.19 vs. 1.04 p = 0.34**
Home physiotherapy (Y vs. N)	15.8%; 95% CI, 10.9-22.3 vs. 28.0%; 95% CI, 17.5-41.7 p = 0.05*	12.3%; 95% CI, 8.0-18.5 vs. 25.5%; 95% CI, 15.2-39.5 p = 0.03*	14.6%; 95% CI, 10.0-21.0 vs. 29.4%; 95% CI, 18.7-43.0 p = 0.02*	14.6%; 95% CI, 10.0-21.0 vs. 29.8%; 95% CI, 18.6-44.0 p = 0.02*	39.3%; 95% CI, 29.8-49.7 vs. 20.5%; 95% CI, 10.8-35.5 p = 0.04*
Use of flutter or shaker (Y vs. N)	12.7%; 95% CI, 7.6-20.6 vs. 29.6%; 95% CI, 19.1-42.8 p = 0.01*	10.1%; 95% CI, 5.6-17.6 vs. 23.1%; 95% CI, (12.5-36.8) p = 0.03*	14.9%; 95% CI, 9.2-23.1 vs. 27.3%; 95% CI, 17.3-40.2 p = 0.06	15.8%; 95% CI, 10.0-24.2 vs. 24.5%; 95% CI, 14.9-37.6 p = 0.19	43.9%; 95% CI, 31.8-56.7 vs. 24.4%; 95% CI, 14.2-38.7 p = 0.04*
Liver disease (Y vs. N)	9.1%; 95% CI, 3.6-21.2 vs. 21.8%; 95% CI, 16.0-28.9 p = 0.08	4.7%; 95% CI, 1.3-15.5 vs. 17.9%; 95% CI, 12.6-24.8 p = 0.03*	11.4%; 95% CI, 4.9-24.0 vs. 22.4%; 95% CI, 11.6-29.6 p = 0.10	9.1%; 95% CI, 3.6-21.2 vs. 20.9%; 95% CI, 15.3-28.2 p = 0.08	56.5%; 95% CI, 36.8-74.4 vs. 29.2%; 95% CI, 21.0-38.9 p = 0.01*
Psychological referral (Y vs. N)	14.6%; 95% CI, 6.9-28.4 vs. 27.6%; 95% CI, 17.7-40.2 p = 0.13	17.1%; 95% CI, 8.5-31.3 vs. 18.5%; 95% CI, 10.4-30.8 p = 0.86	07.5%; 95% CI, 2.6-19.9 vs. 25.9%; 95% CI, 16.3-38.4 p = 0.02*	22.0%; 95% CI, 12.0-36.7 vs. 19.6%; 95% CI, 11.3-31.8 p = 0.78	31.7%; 95% CI, 19.6-46.7 vs. 36.2%; 95% CI, 25.1-49.1 p = 0.64
Shwachman-Kulczycki score, general activity (median)	20 vs. 25 p = 0.007**	20 vs. 25 p = 0.01**	25 vs. 25 p = 0.09	22 vs. 25 p = 0.04**	25 vs. 25 p = 0.50
Shwachman score, radiological findings (median)	15 vs. 15 p = 0.62	16.50 vs. 15 p = 0.91	19 vs. 15 p = 0.15	15 vs. 15 p = 0.88	15 vs. 10 p = 0.03**

T1: time point 1; T2; time point 2; CF: cystic fibrosis; N: no; and Y: yes. ^a^Data expressed as % and 95% CI, except where otherwise indicated. *Chi-square test (p < 0.05). **Mann-Whitney test (p < 0.05).

Symptoms of depression were positively correlated with symptoms of anxiety at both time points. As predicted, depression and anxiety were negatively correlated with rates of treatment adherence (Table 4). 

**Table 4:** Correlations between treatment adherence and psychosocial outcomes.

	Adherence (MMAS-8)	Depression at T1 (PHQ-9)	Depression at T2 (PHQ-9)	Anxiety at T1 (GAD-7)	Anxiety at T2 (GAD-7)
Adherence (MMAS-8)					
Depression at T1(PHQ-9)	−0.42**				
Depression at T2 (PHQ-9)	−0.33**	0.69**			
Anxiety at T1 (GAD-7)	−0.34**	0.69**	0.55**		
Anxiety at T2 (GAD-7)	−0.22*	0.56**	0.72**	0.65**	

T1: time point 1; T2: time point 2; PHQ-9: nine-item Patient Health Questionnaire; GAD-7: seven-item Generalized Anxiety Disorder scale; and MMAS-8: eight-item Morisky Medication Adherence Scale. *Pearson’s correlation coefficient (p < 0.05). **Pearson’s correlation coefficient (p < 0.01).

## DISCUSSION

This is the first study in Latin America to assess mental health in adolescents with CF, measuring the prevalence of depression and anxiety at 14 CF referral centers across Brazil. Using patient-reported outcome measures recommended by the International Committee on Mental Health in Cystic Fibrosis,^11^ we found that 15.4-19.1% of the adolescents with CF in the present study reported elevated symptoms of depression, and 18-19.1% reported elevated symptoms of anxiety. Although the rates of depression in the present study were higher than those reported elsewhere,^3^ they were similar to those reported in other studies using the same screening tools.^1^ ^,^ ^5^ ^,^ ^15^ These results show that one of five adolescents with CF is struggling with mental health challenges. Routine, standardized mental health screening is likely to improve the quality of care for individuals with CF. 

The prevalence of depression in the present study was 19.1% at time point 1 and 15.4% at time point 2. Elevated rates of anxiety were found in 19.1% at time point 1 and in 18% at time point 2. Importantly, these rates are 2-3 times higher than those found in community samples.^23^ These results are similar to those reported in a recent systematic review and meta-analysis of people with CF, with an overall prevalence of 12.7% for depression and 26.2% for anxiety varying across countries.^1^ ^,^ ^2^ ^,^ ^10^ The authors of the aforementioned systematic review and meta-analysis concluded that there is an urgent need for systematic screening and a multidisciplinary approach to addressing mental health concerns in people with CF to reduce complications and the negative impact of depression and anxiety on HRQoL.^3^

A document from the WHO states that Brazil is one of the countries in the world with the highest prevalence of anxiety and depression in the general population (9.3% and 5.8%, respectively).^24^ Graziano et al.^5^ showed that almost half of adolescents with CF reported elevated (mostly mild) depression, although 30% were found to have moderate to severe depression. 

In the present study, depression and anxiety were both significantly correlated with worse treatment adherence. Self-reported adherence was low in the present study, with most of the study participants falling into the poor adherence group (67%). Although CF medications are provided free of charge in the Brazilian Unified Health Care System, the prevalence of poor adherence to treatment was similar to that reported in other studies of CF, as well as in studies of other chronic diseases, in the absence of specific intervention.^23^ ^,^ ^25^ Notably, other barriers affect treatment adherence, including symptoms of depression and anxiety.^26^

Several studies have found associations of depression and anxiety with worse health outcomes. Waters et al.^27^ found that patients with elevated depression and anxiety had worse treatment adherence, lower lung function, lower BMI, more frequent hospitalizations, and worse HRQoL. 

Modi et al.^28^ assessed depression and anxiety in adolescents and young adults with CF and found that 32% reported elevated symptoms; females reported higher levels of anxiety than did males, and mental health scores were positively associated with age (r = 0.28-0.36). In a study comparing CF patients and controls in the 8- to 16-year age bracket in Turkey,^4^ at least one psychiatric comorbidity was found in the group of patients with CF (in 68%, with 46% related to anxiety). Depression was three times more common in people with CF than in controls and was associated with worse disease severity, longer periods of hospitalization, and higher anxiety. In a follow-up study of participants in a landmark epidemiological study,^3^ a single elevated depression screen was associated with a doubling of mortality over five years. Thus, elevated symptoms of depression and anxiety have profound consequences for disease management and health outcomes. These findings reinforce the need for adequate screening, diagnostic confirmation, and mental health care for people with CF.^29^ ^,^ ^30^

Given the high rates of depression and anxiety in adolescents with CF in Brazil, it is critical to note that mental health symptoms were lower in those receiving in-home physiotherapy (a form of support) and psychological referrals and interventions. In the USA, where screening for depression and anxiety is universal across CF centers, educational materials, training courses, and new evidence-based and psychological interventions have been developed. Implementation of mental health screening and treatment have been highly successful, with over 85% of CF centers reporting annual screening of all people with CF ≥ 12 years of age. Assessing mental health in people with CF has also gained greater importance in the context of new, highly effective modulators, which have been shown to have side effects that include worsening mental health, insomnia, and cognitive fogging.^31^ ^-^ ^36^

Our results in Brazil show the importance of annual screening and suggest that tools for evaluating depression, anxiety, and treatment adherence can be enormously useful for early detection and intervention, as well as for reducing stigma. The present study was conducted before the COVID-19 pandemic. Therefore, the rates of depression and anxiety were not a result of the pandemic. In fact, Smith et al. showed that mental health symptoms worsened in adolescents during the COVID-19 pandemic.^37^

The main limitation of our study is the fact that we used a convenience sample rather than collecting data using a randomized sampling strategy. However, given the high number of participating centers, we estimate that our sample size of 218 reflects an accurate profile of adolescents with CF in Brazil, corresponding to 23.1% of the 943 patients included in the Brazilian Cystic Fibrosis Registry in the same age range. It is noteworthy that our sample was larger than those of most of the studies included in a recent systematic review and meta-analysis.^2^

Identifying people with CF at a higher risk of poor treatment adherence and poor disease management is critically important as new medications, such as CFTR modulators, are released. Although CFTR modulators have dramatically improved clinical outcomes, quality of life, and life span, identifying CF patients with mental health challenges will be critical for sustaining adherence in this different era of CF care. To date, studies evaluating mental health outcomes after the introduction of the triple combination of elexacaftor, tezacaftor, and ivacaftor have failed to identify significant changes in the prevalence of depression in treated patients.^38^ ^,^ ^39^ However, the possibility of confounding factors should be highlighted, given the multifactorial aspects of the pathogenesis of mental disorders. 

Mental health screening is a brief, reliable way to assess patient well-being and can easily be incorporated into in-person (e.g., the medical assistant gives out the screening forms once in the clinic room; they can also be given when patients are checked in) or telehealth visits. In a recent survey of people with CF and their families in Europe and the USA, mental health screening was viewed as a positive, supportive intervention on its own. It signaled to patients and families that the CF team was caring and concerned, and it opened the door to a discussion of various brief intervention strategies.^40^ Future directions include conducting a prevalence study of depression, anxiety, and behavior problems in children > 12 years of age and developing a brief checklist of other mental health comorbidities (e.g., substance misuse and procedural anxiety/distress). 

The adolescent patients with CF in the present study showed low rates of treatment adherence, similar to those observed in other cohorts of CF and other chronic diseases, in the absence of specific intervention; an inverse correlation between adherence, depression, and anxiety; an inverse correlation between adherence and age; a positive association between adherence and commitment to care (home physiotherapy and exacerbations treated at home); a direct correlation between depression and anxiety across two time periods; a direct correlation between age, depression, and anxiety; a higher prevalence of depression and anxiety in girls; a lower prevalence of depression and anxiety in patients undergoing home physiotherapy; and a lower prevalence of anxiety in patients receiving psychological care. 

It is well known that anxiety and depression are multifaceted conditions that are not exclusively related to CF. Patients with CF may have other conditions leading to anxiety and depression (e.g., comorbidities and adolescence-related psychosocial factors). Future studies could employ a longitudinal design, including comparison with healthy controls, to allow multivariate analysis in the CF group. These aspects highlight the importance of systematic assessment of psychosocial factors in the life course of individuals with chronic diseases such as CF. 

This is the first multicenter study on mental health in children and adolescents with CF in Brazil. We emphasize the importance of identifying high-risk groups with low treatment adherence, such as those with depression and anxiety. This can contribute to improving the standards of care for CF. 
